# Vitamin A deficiency compromises the barrier function of the retinal pigment epithelium

**DOI:** 10.1093/pnasnexus/pgad167

**Published:** 2023-05-19

**Authors:** Jean Moon, Gao Zhou, Eckhard Jankowsky, Johannes von Lintig

**Affiliations:** Department of Pharmacology, School of Medicine, Case Western Reserve University, Cleveland, OH 44106, USA; Center for RNA Science and Therapeutics, School of Medicine, Case Western Reserve University, Cleveland, OH 44106, USA; Center for RNA Science and Therapeutics, School of Medicine, Case Western Reserve University, Cleveland, OH 44106, USA; Department of Biochemistry, School of Medicine, Case Western Reserve University, Cleveland, OH 44106, USA; Department of Pharmacology, School of Medicine, Case Western Reserve University, Cleveland, OH 44106, USA

**Keywords:** RPE, vitamin A, deficiency, transcriptome, STRA6

## Abstract

A major cause for childhood blindness worldwide is attributed to nutritional vitamin A deficiency. Surprisingly, the molecular basis of the ensuing retinal degeneration has not been well defined. Abundant expression of the retinoid transporter STRA6 in the retinal pigment epithelium (RPE) and homeostatic blood levels of retinol-binding protein delay vitamin A deprivation of the mouse eyes. Hence, genetic dissection of STRA6 makes mice susceptible to nutritional manipulation of ocular retinoid status. We performed RNA-seq analyses and complemented the data with tests of visual physiology, ocular morphology, and retinoid biochemistry to compare eyes with different vitamin A status. Mild ocular vitamin A deficiency decreased transcripts of photoreceptor transduction pathway-related genes and increased transcripts of oxidative stress pathways. The response was associated with impaired visual sensitivity and an accumulation of fluorescent debris in the retina. Severe vitamin A deficiency did not only impair visual perception but also decreased transcripts of genes encoding cell adhesion and cellular junction proteins. This response altered cell morphology, resulted in significant changes in transport pathways of small molecules, and compromised the barrier function of the RPE. Together, our analyses characterize the molecular events underlying nutritional blindness in a novel mouse model and indicate that breakdown of the outer blood–retinal barrier contributes to retinal degeneration and photoreceptor cell death in severe vitamin A deficiency.

**Significance Statement**

Perturbed vitamin A metabolism is a characteristic of many ocular disease states. How the eyes adapt to vitamin A status has not been investigated in molecular detail. Applying RNA-seq and employing STRA6-deficient mice enabled us to determine the ocular transcriptome under various vitamin A supply conditions. STRA6-deficient mice displayed various degrees of vitamin A deficiency whereas wild-type mice maintained homeostasis. By combining data about genetic pathways with imaging, physiological, and biochemical analyses, we integrated transcriptome changes to ocular phenotypes. The studies revealed that under conditions of mild vitamin A deficiency, the eyes suffered from impaired visual physiology and stress. Severe deficiency affected epithelia maintenance and proper tight junction regulation, culminating in a breakdown of the outer blood–retina barrier.

## Introduction

The importance of vitamin A (all-*trans*-retinol) for vision has long been known because deficiency of the nutrient can damage the eyes throughout the life cycle (1). Vitamin A is the precursor for the visual chromophore (retinaldehyde) and all-*trans*-retinoic acid (RA). The 11-*cis*-diastereomer of retinaldehyde forms a Schiff base linkage with the opsin moieties of cone and rod visual pigments. These G protein–coupled receptors mediate phototransduction, the process by which photons are converted into a nerve signal (2). RA is a hormone-like molecule that regulates gene expression through nuclear receptors (3, 4) that are critical for the cell growth, differentiation, and patterning of the eyes (5).

Vitamin A in the blood is delivered to the eyes, thereby supporting retinoid signaling and chromophore synthesis in the eyes. This transport occurs via two overlapping pathways distributing either dietary vitamin A as retinyl esters (REs) in chylomicrons or stored hepatic vitamin A bound to the retinol-binding protein (holo-RBP4) (6). Cellular uptake of vitamin A from these two transport modes is respectively facilitated by lipoprotein lipase or by the RBP4 receptor STRA6 (stimulated by RA6) (7–9). STRA6 is an integral membrane protein of the basolateral membrane of the retinal pigment epithelium (RPE) (10, 11). The protein facilitates the bidirectional transport of vitamin A into RPE cells from circulating holo-RBP4 in a regulated manner (8, 12, 13).

In humans, mutations in the *STRA6* gene cause Matthew–Wood syndrome (MWS) (14–16). MWS is characterized by severe bilateral microphthalmia, often in combination with pulmonary dysplasia, cardiac defects, and diaphragmatic hernia (14). This phenotype is consistent with the pleiotropic roles of RA in embryonic development (17). In contrast, mice deficient for STRA6 or its RBP4 ligand develop eyes and are viable when raised on vitamin A-rich diets (18–20). The eyes of *Stra6* knockout mice do not establish a retinoid pool sufficient to support visual pigment synthesis, resulting in loss of photoreceptor function and retinal degeneration. During adolescence, the eyes of this mouse mutant rely on supply via the chylomicron-dependent delivery pathway of dietary vitamin A (19, 21). Therefore, their ocular vitamin A status can be manipulated by dietary intervention (19, 21). This characteristic makes this mouse mutant a unique model to study nutritional blindness which is a widespread ailment in children worldwide (22).

Seminal work by Dowling and Wald showed that vitamin A deficiency (VAD) in rats affects photoreceptor function and eventually results in retinal degeneration (23). They further demonstrated that RA can fulfill most vitamin A-dependent activities except photoreceptor function (24). Accordingly, chromophore-deficient mouse mutants do not exhibit the retinal degeneration phenotype of VAD eyes (25–27). However, it is not well defined how the eyes adapt to changes in vitamin A status and what events are associated with the retinal degeneration phenotype in severe VAD.

Here, we subjected *Stra6^−/−^* and wild-type (WT) mice to dietary vitamin A deprivation. By this approach, we induced mild and severe ocular VAD in *Stra6^−/−^* mice, as determined by biochemical analysis. Deep sequencing (bulk RNA-seq) analyses revealed the transcriptomes of photoreceptors and RPE under these conditions (28). Based on genotype and diet, we associated the transcriptomes with physiological, histological, and biochemical changes of the eyes. Milder deficiency affected the phototransduction machinery and was associated with an elevated expression of genes involved in oxidative stress pathways. The consequences of severe VAD extended to the structural and functional bases of the outer blood–retina barrier (oBRB). The emerging picture underscores that vitamin A is critical to maintain photoreceptor function and overall visual homeostasis. We identify novel aspects of ocular VAD, specifically the vitamin's role in supporting RPE integrity and function. This role of vitamin A may explain the irreversible retinal degeneration and vision loss in children suffering from prolonged VAD.

## Results

### *Stra6*-deficient eyes model ocular VAD

To investigate how the eyes adapt to vitamin A status, we used *Stra6* knockout mice (18) which exhibit dysregulated ocular retinoid homeostasis (19–21). All mice were bred and raised on a standard rodent diet rich in vitamin A (15 IU/g) to avoid developmental complications. At the time of weaning, WT and *Stra6^−/−^* mice were maintained on a vitamin A sufficient (VAS) or VAD diet, which are custom diets routinely implemented to control vitamin A status in rodents (29). The 4 IU retinol/g existing in the VAS diet is consistent with the recommended vitamin A intake for rodents. Four international units is equivalent to 1.2 mg retinol activity equivalents (RAE) which falls within a range adequate in humans. The VAD diet, a modified AIN-93G diet that is free of vitamin A (0 IU/g), is utilized to restrict vitamin A (29, 30).

**Fig. 1.**
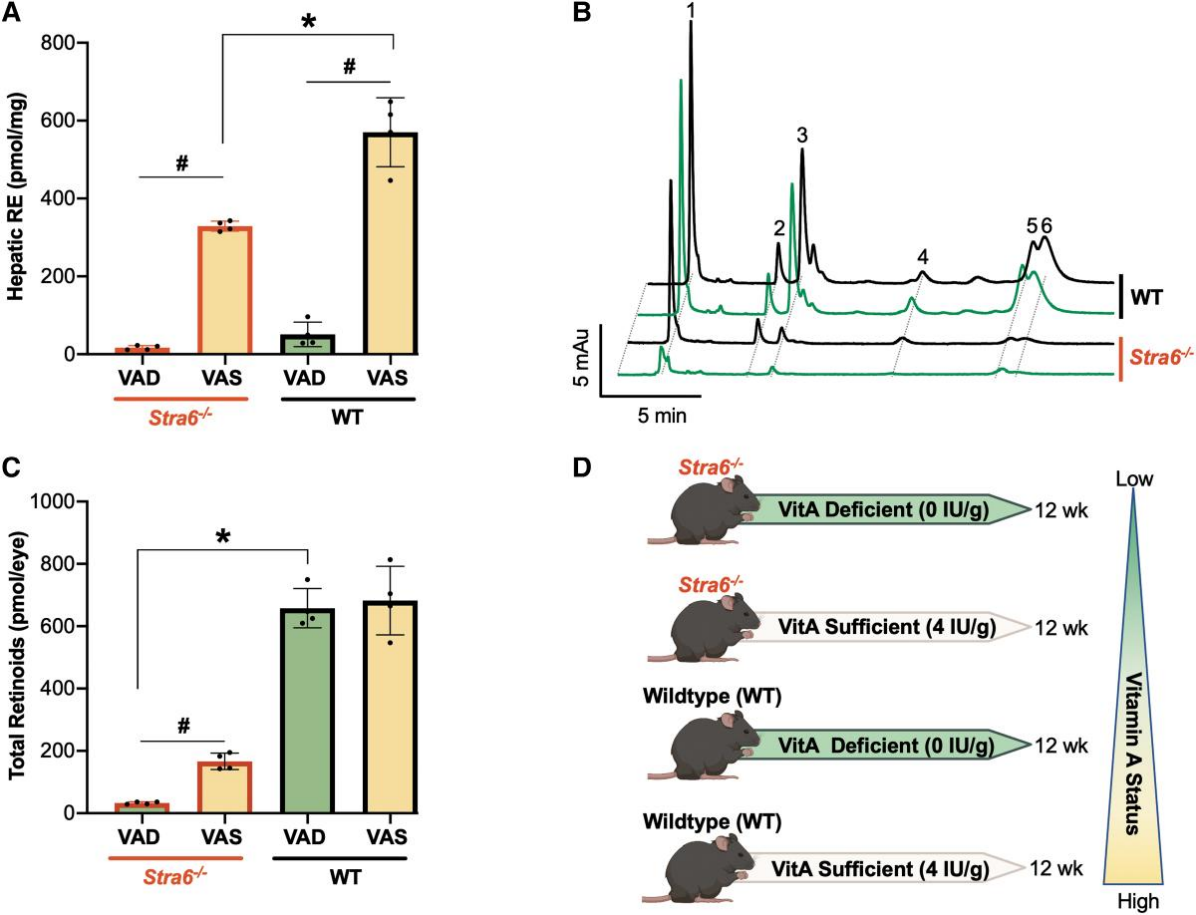
Characterization of retinoid biochemistry. HPLC was used to determine the retinoid content in the livers (A) and eyes (B, C) isolated from WT and *Stra6^−/−^* mice that were maintained on a VAS or VAD diet for a period of 12 weeks. Each group of mice represents varying degrees of ocular VAD (D). RE, retinyl ester; VAD, vitamin A deficient; VAS, vitamin A sufficient; 1, retinyl esters; 2, 11-*cis*-retinal (syn); 3, all-*trans*-retinal (syn); 4, 11-*cis*-retinal (anti); 5, all-*trans*-retinol; 6, all-*trans*-retinal (anti). Values presented as mean ± SD. *P < 0.05 between genotypes, within diet; ^#^*P* < 0.05 VAS vs. VAD, within genotype.

In response to dietary restrictions, hepatic RE stores were significantly depleted on a VAD diet irrespective of genotype, suggesting that the vitamin was redistributed from its major storage site to other tissues. On a VAS diet, the levels of RE in the livers of *Stra6^−/−^* mice were lower than that of WT counterparts (Fig. 1A). High performance liquid chromatography (HPLC) analysis revealed that all visual cycle intermediates exist in all groups of mice (Fig. 1B). The eyes of WT mice, carrying the fully functional *Stra6* allele, were largely unaffected by diet. Ocular retinoid homeostasis was abrogated without STRA6, demonstrated by the significantly reduced concentrations under both dietary conditions. Compared with *Stra6^−/−^* mice on a VAD diet, those on a VAS diet possessed 400% higher retinoid content (Fig. 1B and C). These characteristics reveal that the degree of ocular VAD can be manipulated by a controlled diet in *Stra6^−/−^* mice (Fig. 1D). With such a system, we can ascribe observed changes in VAD *Stra6^−/−^* and VAS *Stra6^−/−^* groups as responses to severe and mild VAD states, respectively.

### Cone and rod phototransduction were compromised in *Stra6^−/−^* mice

**Fig. 2.**
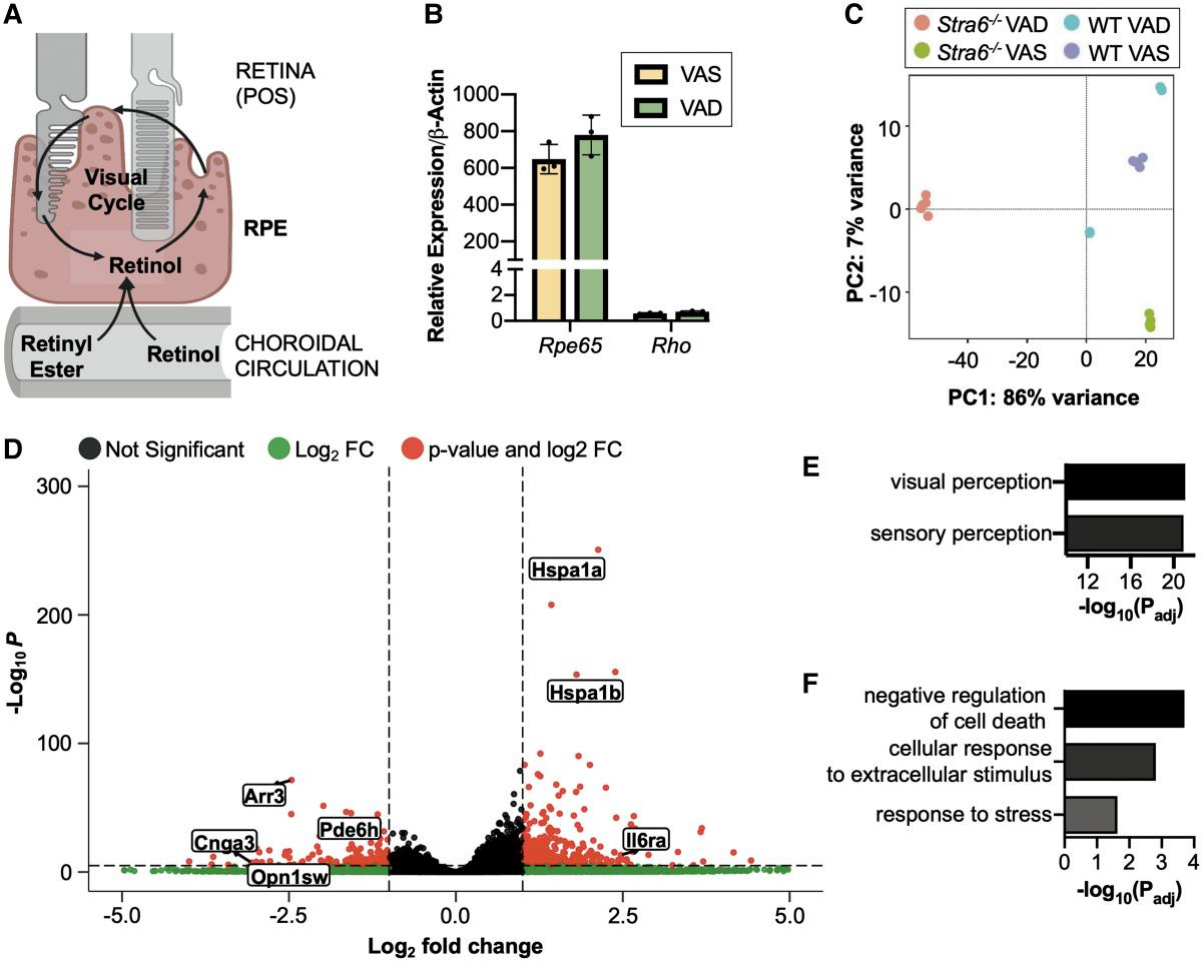
RPE–choroid isolation for bulk RNA-seq. Metabolism and cycling of retinoids exist in the RPE layer which is situated in between the retina and circulation (A). The outer eyecup, consisting of the RPE and choroid, was isolated from mice after a 12-week intervention with either a VAS or VAD diet. *Rpe65* and *Rho* gene expression in the outer cup was assessed using qPCR (B). Plot of the first two components of total gene expression data set (C). Volcano plot of significant genes (D) and GO BP ontology terms from the down-regulated (E) and up-regulated (F) gene sets based on comparing *Stra6^−/−^* VAS relative to WT VAS samples. *n* = 5 mice per group. *Arr3*, arrestin 3; *Cnga3*, cyclic nucleotide-gated channel alpha 3; *Hspa1a*, heat shock protein 1A; *Hspa1b*, heat shock protein 1B; *Il6ra*, interleukin-6 receptor; *Opn1sw*, opsin 1, short-wave-sensitive; *Pde6h*, phosphodiesterase 6H.

The generation and recycling of chromophore occur in the RPE layer (Fig. 2A). While the apical side is adjacent to the photoreceptor outer segments, STRA6 is expressed at the basolateral side of the RPE, facilitating ROL transport from the circulation (8). The RPE tissue acts as a metabolic hub that is an optimal target for assessing the response to varying vitamin A levels. Thus, we collected 40 posterior eye cup tissues (RPE/choroid) to compare changes in the transcriptome between *Stra6^−/−^* and WT mice 12 weeks after placement on a VAS or VAD diet. Treatment with dispase improved separation of the eyecup from the retina (Fig. S1). While the expression of *Rhodopsin* (*Rho*), albeit significantly lower compared with *Retinoid isomerase* (*Rpe65*), indicated cross-contamination in our sample preparation, this allowed us to monitor into the condition of the photoreceptor layer (Fig. 2B).

Both eyecups from each mouse were pooled together as a single biological replicate (*n* = 5 mice per group). All analyses were carried out on the data set comprised of 24,411 genes, and genes with low counts were prefiltered prior to running DESeq2. WT and VAS were used as reference levels for the genotype and diet factors, respectively. To gain insights into the similarity across the conditions, we performed principal component analysis (PCA) (Fig. 2C) and noted *Stra6^−/−^* groups clustered together away from the WT groups. Differential expression (DE) and pathway analyses were carried out on genes with an adjusted *P* < 0.05 and a log_2_ fold change >2 or <−2.

**Fig. 3.**
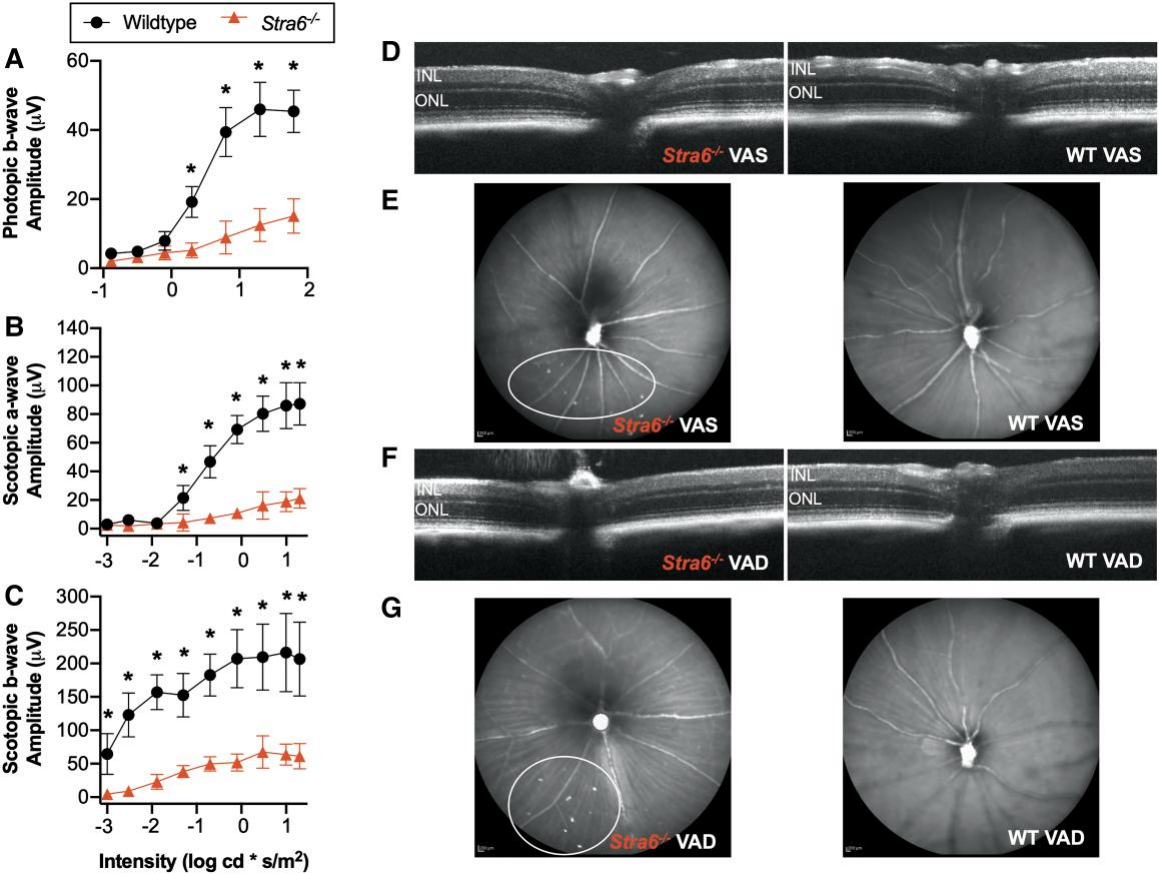
Assessing retina function and morphology. ERG photopic (A) and scotopic (B, C) responses from *Stra6^−/−^* and WT mice (*n* = 4) on a VAS diet for 12 weeks. Representative OCT (D, F) and SLO (E, G) images. Circles indicate autofluorescent spots. INL, inner nuclear layer; ONL, outer nuclear layer. Values presented as mean ± SD. *P < 0.05, unpaired two-tailed *t* test.

We analyzed differentially expressed transcripts in VAS *Stra6^−/−^* samples relative to VAS WT samples. Significantly down-regulated genes included *Cnga3*, *Pde6h*, *Opn1sw*, and *Arr3* which are all involved specifically in the cone phototransduction pathway (Fig. 2D). Furthermore, we observed enrichment for gene ontologies (GO) including visual and sensory perception (fold enrichment = 40) (Fig. 2E). Results from full-field electroretinography supported these findings. Photopic responses, mediated by cone cells, were significantly dampened in *Stra6^−/−^* mice (Fig. 3A). The pattern was similar in dark-adapted, rod cell-driven scotopic a- and b-wave amplitudes (Fig. 3B and C).

### Mild VAD led to inflammation and stress in the RPE

When we analyzed retina morphology by optical coherence tomography (OCT), we did not notice pronounced changes in thickness of the retina layers of *Stra6^−/−^* VAS mice (Figs. 3D and S2). However, scanning laser ophthalmoscope (SLO) images revealed autofluorescent spots in the ventral region of *Stra6^−/−^* that were absent in the retina of WT mice (Fig. 3E and G). Such spots have been previously identified as indication of microglia and macrophage infiltrating and clearing apoptotic photoreceptor cells (31–33). The impact on the retina may be associated with stress in the RPE, as suggested by the enrichment of GO terms cellular response to extracellular stimulus (fold enrichment = 6.8) and response to stress (fold enrichment = 1.6) as well as by the higher transcript levels of *Hspa1a*, *Hspa1b*, and *Angptl4* genes (Fig. 2D and F). Angiopoietin-like proteins are involved in a variety of processes including angiogenesis, vascular permeability, and inflammation, and ANGPTL4 specifically has been implicated in oxidative stress in the RPE (34). Additionally, this was accompanied by the increased expression of *Il6ra*, encoding the interleukin-6 signal transducer receptor, in VAS *Stra6^−/−^* mice.

### VAD affects the retinoid cycle pathway in *Stra6^−/−^* mice

**Fig. 4.**
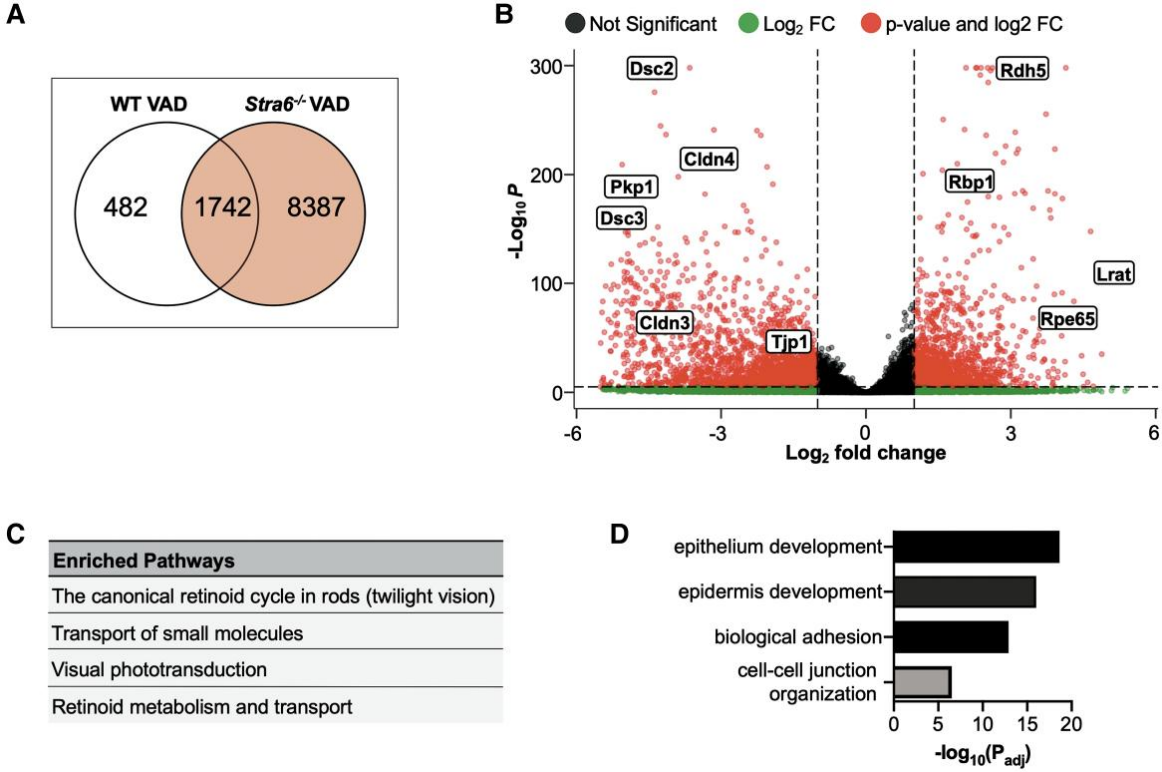
Comparison between *Stra6^−/−^* and WT mice on a VAD diet. Venn diagram representing the overlap of differentially expressed genes between WT and *Stra6^−/−^* VAD samples (A). Volcano plot of significant genes (B). Enriched pathways (C) and GO BP ontology terms from the down-regulated (D) genes comparing *Stra6^−/−^* VAD relative to WT VAD samples. *n* = 5 mice per group. *Cldn3*, claudin 3; *Cldn4*, claudin 4; *Dsc2*, desmocollin 2; *Dsc3*, desmocollin 3; *Lrat*, lecithin–retinol acyltransferase; *Pkp1*, plakophilin 1; *Rdh5*, retinol dehydrogenase 5; *Rbp1*, retinol-binding protein 1, cellular; *Rpe65*, retinal pigment epithelium 65; *Tjp1*, tight junction protein 1.

PCA revealed VAD *Stra6^−/−^* samples were separated from not only WT but also VAS *Stra6^−/−^* samples (Fig. 2C). This accounted for the 86% variance in the first principal component (PC1). We identified DE genes in the VAD *Stra6^−/−^* and WT samples relative to the respective VAS samples. Analyses, using a false discovery rate (FDR) cutoff at 0.05, uncovered 482 genes in VAD WT and 8,387 in VAD *Stra6^−/−^* groups (Fig. 4A). Based on the two analyses, there was an overlap of 1,742 genes in this comparison.

We next performed DE and pathway analyses for VAD *Stra6^−/−^* relative to VAD WT (Fig. 4B–D). For the highly expressed transcripts, we found that the most significantly enriched pathways include the canonical retinoid cycle in rods and retinol metabolism (fold enrichment = 16.4) (Fig. 4C). The genes associated with these pathways are retinol dehydrogenases (*Rdh5* and *Rdh10*), retinoid-binding proteins (*Rbp1* and *Rlbp1*), lecithin retinol acyltransferase (*Lrat*), and retinoid isomerase (*Rpe65*). This suggested that severe VAD results in a transcriptional up-regulation of retinoid cycle genes or a selective stabilization of their transcripts.

### Consequences of VAD on structure of the outer BRB

The top biological processes (BP) that are enriched in VAD *Stra6^−/−^* compared with VAD WT include epithelium development, cell adhesion, and cell–cell junction organization (fold enrichment = 2) (Fig. 4D). The genes that are down-regulated in these processes are claudins (*Cldn3* and *Cldn4*), plakophilin-1 (*Pkp1*), and desmocollins (*Dsc2* and *Dsc3*). These different classes encode cell junction proteins in the RPE (Fig. 4B). The desmocollins are cadherins which, together with plakophilins, reside within desmosomal plaques at the basolateral side of the epithelium (35). Claudin proteins make up part of the tight junctions which confer selective barrier function of the oBRB. The effect of the diet on the *Stra6* genotype, as seen by differential analysis between VAD and VAS *Stra6^−/−^*, reiterates the effect on cell adhesion (Fig. S4A). The significantly down-regulated *Tjp1* gene encodes one of the major RPE tight junction proteins zonula occludens (ZO)-1. ZO-1, together with occludins, contributes to forming an intact barrier and is integral for preserving RPE polarity (35). These results collectively suggest that vitamin A restriction significantly affects the structural integrity of the oBRB.

In accordance with the ocular retinoids (Fig. 1B and C), retina morphology (Figs. 3F and S2), and electroretinography (ERG) responses (Fig. S3), we did not note considerable changes in the VAD WT group. However, of the gene set, transcript levels of *Thbs4* which encodes the extracellular matrix (ECM) protein Thrombospondin-4 (TSP-4) were significantly higher. Elevated TSP-4 protein expression in injured tissues has been associated with mechanisms of ECM remodeling (36). Similarly, the ECM organization pathway (fold enrichment = 2.6) was significantly enriched in VAD *Stra6^−/−^* (Fig. S4B). *Comp* and *Mmp12*, encoding additional ECM remodeling proteins, transcripts were abundant, implying alterations in the structural support between the RPE, Bruch's membrane, and choroid.

### Severe VAD disrupted oBRB maintenance and permeability

**Fig. 5.**
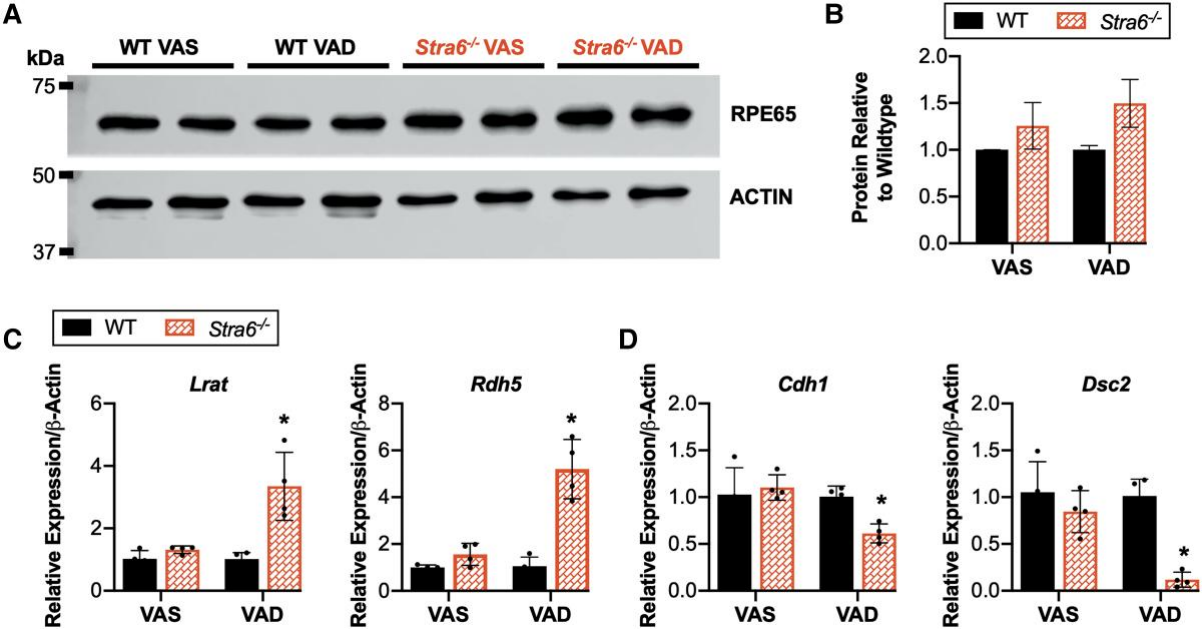
Validation by western blot and qPCR. The protein levels of RPE65 were assessed by western blot analysis (A). The loading control β-actin was used for normalization, and the corresponding expression was quantified relative to WT genotype (B). RPE–choroid tissue from four mice (*n* = 4) were isolated, and each lane represents a unique pool from two mice. The RPE expresses visual cycle genes *Lrat* and *Rdh5* which have been quantified by PCR (C). Relative quantification of mRNA for *Cdh1* and *Dsc2* which encode for intercellular junction proteins (D). *Cdh1*, cadherin 1; *Dsc2*, desmocollin 2; *Lrat*, lecithin–retinol acyltransferase; *Rdh5*, retinol dehydrogenase 5; *Rpe65*, retinal pigment epithelium 65. Values presented as mean ± SD. *P < 0.05 between genotypes, within diet.

**Fig. 6.**
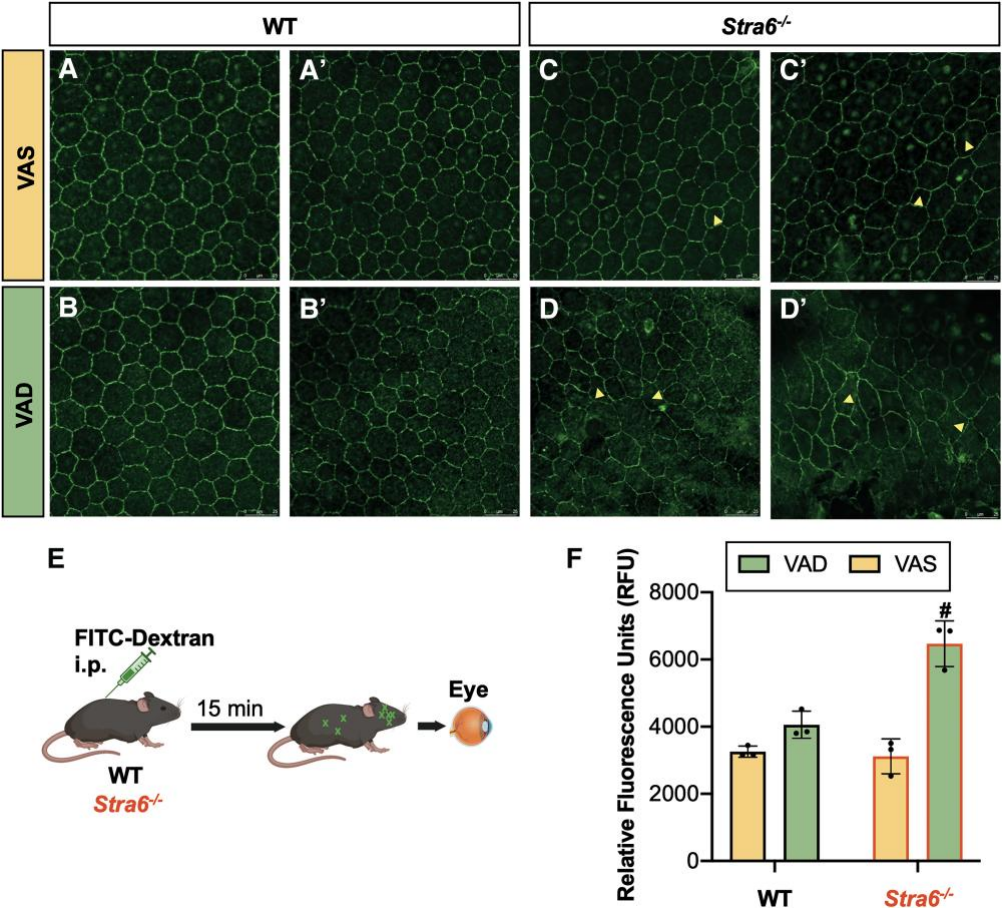
Examining oBRB morphology and barrier function. RPE flatmounts were prepared and stained for ZO-1 (A–D). The adjacent panels for each group marked by (’) represent an independent mouse RPE biological replicate. Scheme of FITC–dextran injections to assess permeability (E). Fluorescence was measured in retina tissue using a spectrophotometer (F). Sham control (phosphate buffered saline [PBS] alone) was used to subtract background autofluorescence. Arrow symbols indicate irregularities in RPE cell morphology. Values presented as mean ± SD. ^#^*P* < 0.05 between diets, within genotype.

The modest elevated RPE65 protein expression in RPE tissue from mice on a VAD diet (Figs. 5A and B and S5) correlates with the enrichment of the retinoid cycle pathway in VAD *Stra6^−/−^* samples (Figs. 4C and S4B). Quantitative PCR results further supported the increase in visual cycle gene expression (Fig. 5C) as well as the decrease in expression of genes encoding cellular junction proteins. Epithelial marker E-cadherin *Cdh1* and desmosomal cadherin *Dsc2* were down-regulated in *Stra6^−/−^* compared with WT (Fig. 5D). Intercellular adhesion proteins serve to preserve tissue integrity and RPE structure, and therefore, we prepared whole RPE flatmounts stained with ZO-1 to assess overall morphology. RPE tissue consists of a distinct single layer of cells that are hexagonally packed, as illustrated in the WT VAS and VAD groups (Fig. 6A and B). However, anomalies were predominantly detected in STRA6-deficient RPE tissues. Under the VAS conditions, a few epithelial cells no longer conformed to this pattern, whereas considerable variability in cell shape and size occurred with VAD (Fig. 6C and D).

Considering this shift in genes associated to the structural makeup of the oBRB, we investigated whether the transcriptional changes translated to a loss of barrier function. To evaluate if the oBRB has the capacity to properly filter macromolecules, we tested permeability of 10 kDa fluorescein isothiocyanate (FITC)–dextran (Fig. 6E). FITC–dextran was injected i.p. to WT and *Stra6^−/−^* mice 12 weeks after the dietary intervention, and eye samples were collected. To ensure we were accounting for FITC–dextran which crossed the barrier rather than that which may be adhering to the exterior surface of the eye, fluorescence was measured from isolated retina tissue. As shown in Fig. 6F, the relative fluorescence in VAD WT mice was slightly higher, albeit not significant, compared with VAS WT mice. However, the significantly elevated fluorescence intensity of the *Stra6^−/−^* VAD group suggested increased permeability and a compromised oBRB.

### Up-regulation of transporters in the solute carrier superfamily and ATPase subunits

One of the barrier functions is to control the exchange of solutes and nutrients between the circulation and the retina. There was an enrichment in the ion and transmembrane transport terms (fold enrichment = 3.5). The transcripts for numerous genes encoding transporters belonging to the SLC were elevated in *Stra6^−/−^* VAD samples (Fig. S6). *Slc2a1* encodes glucose transporter 1 (GLUT1) which transports glucose to the retina where it is utilized as an energy source. *Slc6a* genes encode for the creatine transporter (CRT) protein for uptake of creatine needed to maintain ATP levels in the retina (37). Interestingly, the *Atp1b1* gene, encoding the β subunit of the Na,K-ATPase, was also elevated. Previous studies suggest that Na,K-ATPase activity is involved in the structure of tight junctions. Alterations in the permeability and ion homeostasis have been shown to affect the levels of Na,K-ATPase expression at either the apical or basolateral side of the RPE (38). Taken together, these results strengthen the observed link between VAD and a loss of oBRB function which may result in retinal degeneration.

## Discussion

VAD is a serious public health concern, and an estimated 200 million children are at risk of VAD disorders (22, 39–41). The eyes are particularly vulnerable to this ailment (42). In its milder form, ocular VAD causes reversible night blindness. Clinically, the deficiency manifests as xerophthalmia with conjunctival xerosis and Bitot spots and eventually leads to corneal xerosis, corneal ulceration, keratomalacia, and retinal dystrophies (22, 42). While the significance of VAD for the front of the eyes is well defined, the pathological consequences for the back of the eyes are less well studied (43).

More than 60 years ago, Dowling and Wald described the consequences of VAD in rat retina (23). Initially, the depletion of liver stores of the vitamin A led to a decrease in blood ROL levels. In the retina, rod visual pigment (Rho) declined first before the opsin protein vanished in photoreceptors. The decline of Rho was mirrored in abnormal ERG responses and histological alteration of the retina (23). Since mice only develop ocular VAD when subjected to months of dietary intervention with vitamin A-free diet (27), follow-up studies were conducted in *Rpe65^−/−^* mice (44). These chromophore-deficient mice displayed activation of sensory transduction by unliganded opsin and slow degeneration of rods (45, 46), whereas cone photoreceptors died rapidly (47). It has been previously reported that this condition differs from nutritional VAD in mice (27). In fact, severe VAD is associated with irreversible retina degeneration (48). To better understand the pathology, we herein investigated adaptive responses to vitamin A deprivation. We identified impacted pathways in an unbiased approach from the RPE–choroid tissue, which separates the retina from the choroidal circulation. The photoreceptor layer is avascular, and therefore, nutrients from the choroid are provided through the RPE layer (49, 50). We developed a method to simulate mild and severe deficient states in mice. RNA-seq analyses have revealed effects not only in the retinoid cycle and phototransduction cascade but also epithelia structural maintenance. These findings are consistent with two essential roles of the RPE–choroid layers, production of 11-*cis*-retinal and oBRB function (51–55).

What is the consequence of restricting vitamin A in the diet? The eyes maintain critical processes using mechanisms established to avoid vulnerability to vitamin A status (1). Total ocular retinoid levels were unaffected in WT mice but significantly reduced when STRA6, integral to the homeostatic system, is not expressed. *Stra6^−/−^* mice on the VAS diet experienced a ~75% decrease, while those on a VAD diet possessed trace amounts in the ocular tissue (Fig. 1B and C). Neither preformed vitamin A carried by chylomicrons nor retinol diffusing passively can fully substitute for STRA6-mediated uptake. Both mild and severe deficiency resulted in a decrease of scotopic and photopic vision (Figs. 3A and S3). This was accompanied by a significant down-regulation of genes encoding proteins orchestrating cone phototransduction (Fig. 2D). In a chromophore-deprived photoreceptor, cone opsins are mistrafficked and cone-specific proteins such as PDE6 protein levels are reduced (47, 56). Moreover, ligand binding improves opsin stability and pigment trafficking to cone outer segments. Rods outnumber cones in the retina, so competition for limited chromophore is unfavorable for cones (57). Thus, mild VAD shows many characteristics described in chromophore-deficient mouse eyes.

RPE cells are joined together by tight and adherens junctions near the apical microvilli as well as desmosomes close to the basolateral membrane (52, 58, 59). Optimal ion and nutrient homeostasis in the subretinal space are necessary for preserving photoreceptor excitability and overall retinal health (60–62). A compromised oBRB triggers retinal degeneration, and retinoid signaling is required for development of this structure (35, 63). However, the relationship between vitamin A status and the oBRB has not been well established. *Stra6^−/−^* mice exhibiting severe VAD showed a down-regulation of transcripts of genes encoding proteins that span all three junctions (Fig. 4). Epithelial and endothelial cell layers have tight junctions generating selective barriers that enable tissue-specific activity as seen in the skin, intestine, brain, and eye (64, 65). RA is a vitamin A metabolite whose signaling has been implicated in the maintenance of tissue–blood barriers by altering expression of tight junction proteins (63, 66, 67). The BRB is analogous to the blood–brain barrier (BBB), and the importance of RA signaling has been emphasized during the developmental stages. Interestingly, prior research has demonstrated RA induces BBB genes such as tight junction proteins ZO-1 and cadherins (68). The exact influence of RA on the BBB and a variety of epithelia is inconclusive, as in vitro and in vivo testing has presented results showing both positive and negative RA effects (69–72).

The BRB consists of both outer and inner components, and the integrity of the inner BRB, lining the retinal vasculature, is vulnerable to reduced RA signaling (63). A similar phenomenon was supported in our studies of the oBRB. Phenotypically, RPE from WT mice followed a repetitive, hexagonal shape. In contrast, the RPE cells varied in shape and size in *Stra6^−/−^* mice on a VAD diet. A loss of structural integrity translated to increased permeability to macromolecules (FITC–dextran) (Fig. 6). Our gene set indicated a down-regulation of genes encoding alcohol and aldehyde dehydrogenases involved in RA metabolism. The role of vitamin A in maintaining the oBRB is novel and has implications beyond the physical integrity of the ocular tissue. Tight junctions are multiprotein complexes constituting of transmembrane, peripheral, and cytoplasmic proteins. Many proteins associated with tight junctions act as adaptors to stabilize junctions or scaffolds to recruit proteins for intracellular signaling. Additionally, tight junctions demarcate boundaries between the apical and basolateral membranes, and the polarized distribution of transporters and channels is important for supporting the adjacent photoreceptors (73–75).

Together, our study revealed that ocular consequences of nutritional VAD are attributed to not only chromophore deficiency but also compromised retinoid signaling in the RPE. Damage to the RPE causes other retinal diseases such as age-related macular degeneration (76). How retinoid homeostasis conferred by STRA6 or oBRB transcriptional alterations is associated to the pathophysiology of such diseases warrants further investigations. A better understanding of the role of vitamin A at the oBRB will aid in the development of intervention strategies for children suffering from severe VAD eventually leading to retinal degeneration and vision loss as well as for other blinding diseases.

## Materials and methods

### Animal care and use

The use of mice in this study was approved by the Case Western Reserve University (CWRU) Institutional Animal Care and Use Committee. *Stra6^−/−^* mice on a C57BL/6J genetic background were generated in the vivarium at CWRU as previously described. WT mice were obtained from the Jackson Laboratory. All mice were bred and raised on a standard chow diet containing ∼15,000 IU vitamin A/kg diet (Prolab RMH 3000, LabDiet, St. Louis, MO, USA) with ad libitum access to food and water on a 12:12 h light/dark cycle. After weaning, mice were supplemented with a VAS (4,000 IU vitamin A, retinyl acetate) or VAD (0 IU vitamin A) diet. All mice were maintained on either diet for 12 weeks which is an established timepoint when vitamin A stores are depleted in mice (21). The diets were prepared by Research Diets (New Brunswick, NJ, USA). At the end of the dietary intervention, mice were anesthetized by using a ketamine (60 mg/kg) and xylazine (5 mg/kg) cocktail. Mice were transcardially perfused with PBS and sacrificed by cervical dislocation. Tissues were immediately harvested for analysis or snap frozen in liquid nitrogen and stored at −80°C until further use (77,78).

### HPLC retinoid analysis

Preparation and analyses of retinoid concentrations were performed as previously described (20, 21, 77, 78). For the liver, 10 mg of tissue was homogenized in 200 μL PBS. One entire eyecup was homogenized in 200 *μ*L hydroxylamine (2 m, pH 6.8). Retinoids were extracted twice using a mixture of 200 *μ*L methanol, 400 *μ*L acetone, and 500 µL hexane added to the tissue homogenates. A normal-phase Zorbax SIL (5 *μ*m, 4.6 × 150 mm) column was used for HPLC analysis. Chromatographic separation was achieved by isocratic flow of 10% ethyl acetate/90% hexanes. To quantify the molar amounts of retinoids, the HPLC was previously calibrated with synthetic standard compounds.

### RPE eyecup isolation and RNA extraction

Eyes were enucleated, and muscle and connective tissue were removed, followed by the removal of the anterior segment and vitreous. The eyecup was incubated with 2% dispase for 15 min in 37°C. The neural retina was carefully peeled off while limiting the extent of disturbing the RPE layer, and the remaining RPE–choroid tissue was rinsed with PBS. The RPE–choroid tissues from two eyecups were pooled together for RNA isolation. Tissue was immediately homogenized in 500 *μ*L of TRIzol, 100 *μ*L of chloroform was added, and the samples were centrifuged at 12,000 × *g* for 15 min at 4°C. The aqueous phase was collected, and 500 *μ*L of 70% ethanol was added. This mixture was added directly to a RNeasy mini column (Qiagen). RNA extraction was completed using the RNeasy Plus Mini Kit.

### cDNA library preparation and RNA sequencing

Quantitation and sample integrity and purity were tested using the Nanodrop and Agilent 2100 for quality control. Only samples with A260/A280 ratios >1.8–2 were submitted for further processing at Novogene Genomics Technology. For library construction, mRNA was enriched using oligo(dT) beads, fragmented randomly, and then cDNA was synthesized using random hexamer primers, dNTPs, RNase H, and DNA polymerase I. Following terminal repair, A ligation, and sequencing adaptor ligation, the double-stranded cDNA library was completed through size selection and PCR enrichment. The Illumina NovaSeq platform was used to sequence samples using paired-end sequencing (2 × 150 bp). The data discussed in this publication have been deposited in NCBI’s Gene Expression Omnibus and are accessible through GEO Series accession number GSE219252 (https://www.ncbi.nlm.nih.gov/geo/query/acc.cgi?acc=GSE219252) (28).

### Mapping, gene-level analysis, and GO analysis

The samples have an average 26.2 million 150 bp long paired-end reads. Fastqc (version 0.11.2) was used for sequencing quality assessment. Multiqc (version 1.5) was used to aggregate results into a single report. Reads were then aligned to GRCm38/mm10 genome (UCSC genome source, 2011) using STAR (version 2.5.3a). An average of 87.4% of reads was uniquely mapped to the reference genome. The correlated annotation file (GTF) was applied and the gene expression level was determined by RSEM (version 1.2.30) quantification. Lowly expressed genes (counts less than five) were filtered. From the 18,691 remaining genes, analysis was carried out using DESeq2, which normalizes for sequencing depth differences (79). We were investigating how diet and the *Stra6* mutation influence gene expression, and we opted in using a multifactor design. VAS and WT were set as the reference levels for comparisons. Significant genes were determined by the adjusted *P* < 0.05. The threshold was set to include all genes with a log_2_ fold change >|2|. GO analysis, enrichment of BP terms, and pathway analysis were performed using the g:GOSt tool from g:Profiler (80, 81). Overrepresentation of these processes was assessed by fold enrichment that was computed by comparing the relative frequency of input genes in the samples to the frequency of genes annotated to the term or pathway in the background (universe).

### qRT-PCR

Total RNA was extracted from RPE eyecups using the TRIzol method (Invitrogen, Carlsbad, CA). The Nano drop ND-1000 spectrophotometer (Thermo Fisher Scientific, Waltham, MA, USA) was used to quantify RNA, and cDNA was generated using the High Capacity RNA to cDNA kit (Applied Biosystems, Thermo Fisher Scientific). RT-PCR was carried out using the TaqMan Master Mix (Applied Biosystems, Thermo Fisher Scientific) and the primers (Applied Biosystems) to amplify *β-actin* (Mm02619580), *Cdh1* (Mm00516355), *Dsc2* (Mm00516355), *Lrat* (Mm00469972), and *Rdh5* (Mm07299950). Based on the comparative delta-Ct method, gene expression levels were normalized using the housekeeping gene *β-actin*.

### Immunoblotting

RPE65 protein levels were assessed in the RPE–choroid tissue isolated from the mouse eyes. Tissue samples were homogenized in T-PER Tissue Protein Extraction Reagent (Thermo Scientific) solution with 2 mm phenylmethylsulfonyl fluoride (PMSF). After 30-min incubation on ice, the supernatant was isolated after centrifugation (15 min, 10,000 × *g* at 4°C) and stored at −80°C until further use. Protein concentrations were determined using the BCA protein assay (Thermo Fisher Scientific). Forty micrograms of protein lysates was mixed with 2X loading buffer (1 m Tris-HCl, pH 6.8, 1 m dithiothreitol (DTT), and 10% sodium dodecyl sulfate [SDS]) and loaded on Sodium dodecyl-sulfate polyacrylamide gel electrophoresis (SDS–PAGE) gels (12%). The protein was then transferred to polyvinylidene difluoride (PVDF) membranes (Bio-Rad). Membranes were blocked using 5% (*w*/*v*) nonfat dry milk dissolved in Tris-buffered saline containing 0.01% Tween-20 for 1 h at room temperature. The blots were washed and incubated overnight at 4°C with the appropriate primary antibody. As a loading control, β-actin antiserum (Cell Signaling, Boston, MA) was used at a dilution of 1:2,000. The antibody to RPE65 (noncommercial) was used to probe the blots. Goat antimouse IgG (Promega, Madison, WI) was employed as a secondary antibody (1:5,000) and incubated for 1 h at room temperature. All antibodies were diluted in Tris-buffered saline containing 0.01% Tween-20. The Odyssey Imaging System (LI-COR Biosciences) was used to scan western blots with chemiluminescence.

### Histology

Following PBS perfusion, the eyes were isolated from mice and fixed in 4% paraformaldehyde overnight at room temperature. The Visual Sciences Research Center Histology Core at Case Western Reserve University conducted paraffin embedding, retinal sectioning (10 μm), and hematoxylin and eosin (HE) staining. The HE-stained sections were imaged using the Leica DM600 microscope (77).

### The RPE flatmount and immunostaining

Following euthanasia, the eyes were immediately harvested and fixed with 4% paraformaldehyde for 1 h. The cornea, lens, and vitreous were removed, and radial incisions were made to flatten the remaining eyecup. The RPE layer was separated from the retinal tissue. The RPE flatmounts were incubated with 0.5% Triton X-100 in PBS (30 min) for permeabilization and then with ZO-1 polyclonal primary antibody (1:100; Thermo Fisher Scientific, 61-7300) overnight at 4°C. The flatmounts were washed with PBST (0.1% Triton X-100 in PBS). Alexa Fluor 488 was used as the secondary antibody (1:500; 2 h room temperature). The flatmounts were washed with PBST and then PBS before mounting on microscope slides. Images were acquired using the Leica Hyvolution SP8 confocal microscope. The argon laser (excitation 488 nm) was used with a ×63 C-Apochromat, NA, 1.4-O objective.

### Spectral domain OCT and SLO imaging

One percent tropicamide (Bausch and Lomb, Tampa, FL) eye drops were applied to dilate the pupils prior to the eye examination. Mice were anesthetized intraperitoneally with a ketamine/xylazine rodent cocktail. The optic nerve was positioned in the center before acquiring spectral domain OCT (SD-OCT, Bioptigen) images. B-scan images were acquired and averaged using the Bioptigen software (20). Retinal changes between treatment groups were assessed by measuring the thickness of the outer retina layers (including photoreceptor outer nuclear layer and inner/outer segments) 0.25–0.75 mm from the optic nerve head. The thickness values were averaged from retinas of four mice in each experimental group. Mouse fundus images were collected using the confocal SLO (SLO Spectralis HRA2, Heidelberg Engineering, Heidelberg, Germany). To obtain evenly illuminated fundus images, the fundus camera was aligned to the pupil using the near-infrared reflective laser (820 nm) (20, 77).

### Electroretinography

Mice were dark adapted overnight prior to examination. One percent tropicamide (Bausch and Lomb, Tampa, FL) was used to dilate the pupils, and mice were anesthetized using a ketamine/xylazine rodent cocktail. Mice were placed on a heating pad throughout the recording session to keep the body temperature at 37°C. Ag–AgCl electrodes were placed on the surface of the cornea (Diagnosys Celeris, Lowell, MA). For dark-adapted ERG responses, mice were exposed to 10 steps of a white light, flash stimulus ranging from 0.001 to 20 cd s × m^−2^. Interstimulus intervals were set at 4 s for low-luminance flashes and 90 s for the highest stimulus. For cone ERG responses, mice were exposed to a steady adapting light field for 7 min, and waveforms were recorded with strobe-flash stimuli (0.32 to 63 cd s × m^−2^) superimposed on the adapting field. Amplitude values were averaged between left and right eyes (20, 77).

### FITC–dextran injections

The permeability of the oBRB was assessed using a method adapted from an assay measuring BBB permeability (82). After *Stra6^−/−^* and WT mice were supplemented with either a VAS or VAD diet, three mice were injected intraperitoneally with 10 and 40 kDa FITC–dextran or PBS (sham). After 5 min, the mice were injected with ketamine–xylazine. The mice were perfused with PBS. The eyes were then immediately enucleated, and the retina tissue layer was pooled from each mouse for further processing. The tissue was homogenized in cold PBS, centrifuged at 10,000 × *g* for 20 min at 4°C, and the supernatant was transferred to a new tube. Fluorescence was measured in retina tissue supernatants using the FlexStation 3 Multi-Mode Microplate Reader (Molecular Devices). Fluorescence measurements acquired from the sham control group were used to account for autofluorescence and to subtract background. To avoid photobleaching, the samples were shielded from light at all stages.

### Statistical analysis

Unpaired *t* test was used when comparing values between genotype (WT vs. *Stra6^−/−^*) for ERG results. Two-way ANOVA was used when the observed effect is influenced by both variables, diet and genotype. The Tukey method was carried out for post hoc multiple comparisons. An alpha level of *P* < 0.05 was considered significant.

## Supplementary Material

pgad167_Supplementary_Data

## Data Availability

The data that support findings in this publication are available in NCBI’s Gene Expression Omnibus (28) and are accessible through GEO Series accession number GSE219252 (https://www.ncbi.nlm.nih.gov/geo/query/acc.cgi?acc=GSE219252).
