# Supplementary material for: Vitamin A deficiency compromises the barrier function of the retinal pigment epithelium
Source: PNAS Nexus. 2023 May 19;2(6):pgad167. doi: 10.1093/pnasnexus/pgad167 (PMC10235913; doi:10.1093/pnasnexus/pgad167)
Supplement: pgad167_Supplementary_Data [file pgad167_supplementary_data.pdf]

PNAS nexus

Supplementary Information for

Vitamin A deficiency compromises the barrier function of the retinal pigment epithelium.

Jean Moon[a], Gao Zhou[b], Eckhard Jankowsky[b,c], and Johannes von Lintig[a,*#]

[a] Department of Pharmacology, School of Medicine, Case Western Reserve University, Cleveland, OH, 44106, USA

[b] Center for RNA Science and Therapeutics, School of Medicine, Case Western Reserve University, Cleveland, OH, 44106, USA

[c] Department of Biochemistry, School of Medicine, Case Western Reserve University, Cleveland, OH, 44106, USA

* To whom correspondence should be addressed. Department of Pharmacology (W341), School of Medicine, Case Western Reserve University, 10900 Euclid Avenue, Cleveland, OH 44106, USA. Tel: +1 2163683528; Fax: +1 2163681300.

Email: johannes.vonlintig@case.edu.

This PDF file includes:

Figures S1 to S6

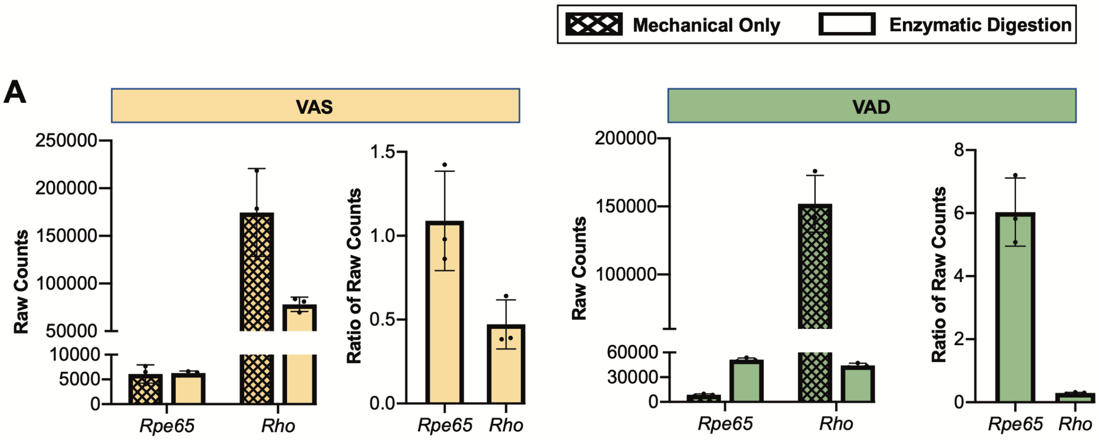

Figure S1. Enrichment of RPE tissue in isolated eyecup. After enucleation, the eyecup was isolated with (enzymatic digestion) or without (mechanical only) pretreatment with dispase. Count data, after normalization, for *Rpe65* and *Rho* genes was assessed to examine which method would increase yield of the RPE layer while minimizing contamination with the retina layer (*A*).

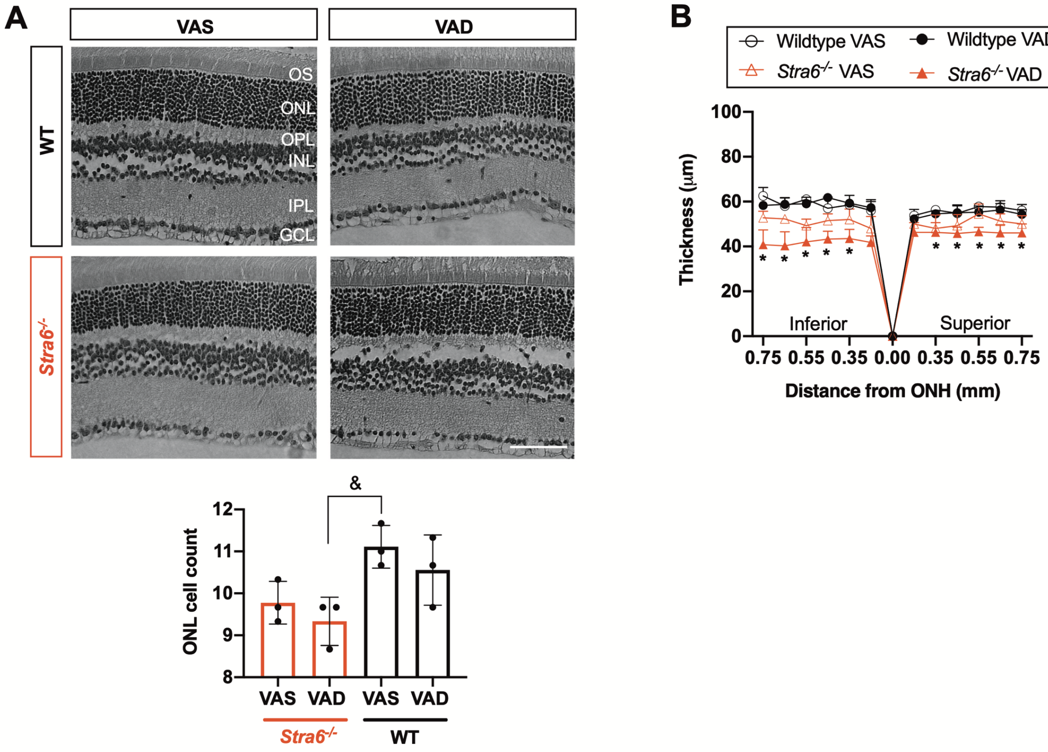

Figure S2. Assessment of retina morphology. (*A*) H&E staining of paraffin sections of retina prepared from eyes isolated from WT and *Stra6*$^{-/-}$ mice, maintained on a VAS or VAD diet, and the counts of number of nuclei in the ONL. Counts were collected from three unique regions of the retina (*n*=3) and averaged. Scale bar, 50 µm. (*B*) Measurements of photoreceptor layer thickness collected from OCT images (*n*=4). GCL: ganglion cell layer; IPL: inner plexiform layer; INL: inner nuclear layer; ONL: outer nuclear layer; ONH: optic nerve head; OPL: outer plexiform layer; OS: outer segment. * $p<0.05$ between *Stra6*$^{-/-}$ and WT VAD. & $p<0.05$ between *Stra6*$^{-/-}$ VAD and WT VAS

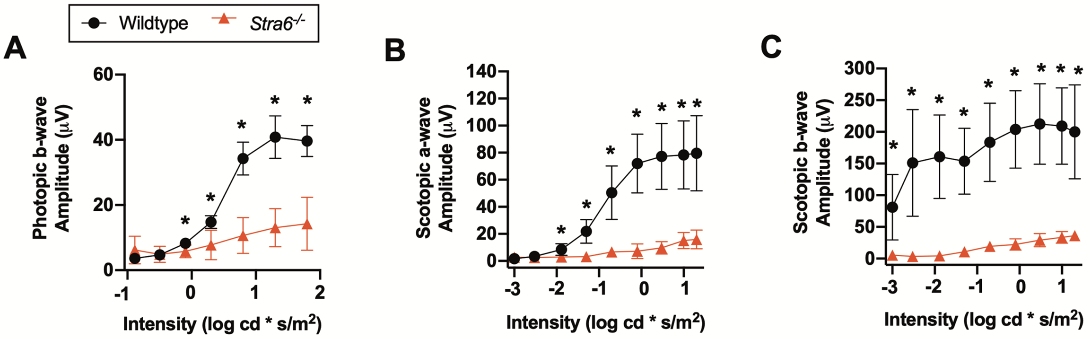

Figure S3. Visual responses from mice on a vitamin A deficient diet. Photopic (*C*) and scotopic (*B*-*C*) ERG responses were recorded from WT and *Stra6*$^{-/-}$ mice (*n*=4) that were on a VAD diet for 12 wk.

A

Enriched Pathways	p_{adj}	gene symbols
Apoptotic cleavage of cell adhesion proteins	3.487×10^{-4}	*Dsp, Pkp1, Dsg1a, Dsg2, Tjp1*
Cell junction organization	2.165×10^{-3}	*Krt14, Plec, Itga6, Itgb4, Lamb3, Krt5, Jup, Lama3, F11r, Prkcl*
Apoptotic cleavage of cellular proteins	3.703×10^{-2}	*Dsp, Plec, Pkp1, Dsg1a, Dsg2*

B

Enriched Pathways	p_{adj}	gene symbols
SLC-mediated transmembrane transport	1.882×10^{-4}	*Slc2a12, Slc6a20a, Slc7a10, Slc13a3*
Transport of small molecules	1.321×10^{-2}	*Trf*
Extracellular matrix organization	1.596×10^{-2}	*Jam3, Mmp12, Loxl4, Lum, Dcn, Col11a1, Matn3, Emilin3, Comp*
The canonical retinoid cycle in rods (twilight vision)	2.491×10^{-2}	*Lrat, Rpe65, Rlbp1, Rdh10*
Transport of organic anions	4.296×10^{-2}	*Slco1c1, Slco1a4*

Figure S4. Differential expression analysis between VAD and VAS *Stra6*$^{-/-}$ samples. (A) The top pathways in downregulated and upregulated (*B*) genes observed using Reactome pathway analysis.

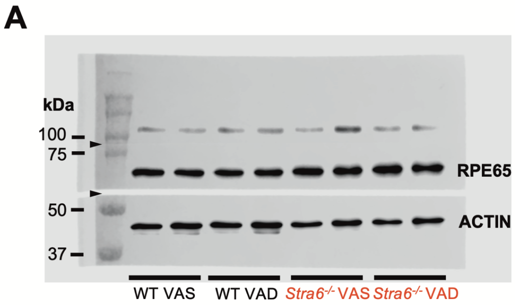

Figure S5. Full Western blot (corresponding to Figure 5A). Membrane was cut, as indicated by arrowheads, and probed for RPE65 and ACTIN (*A*).

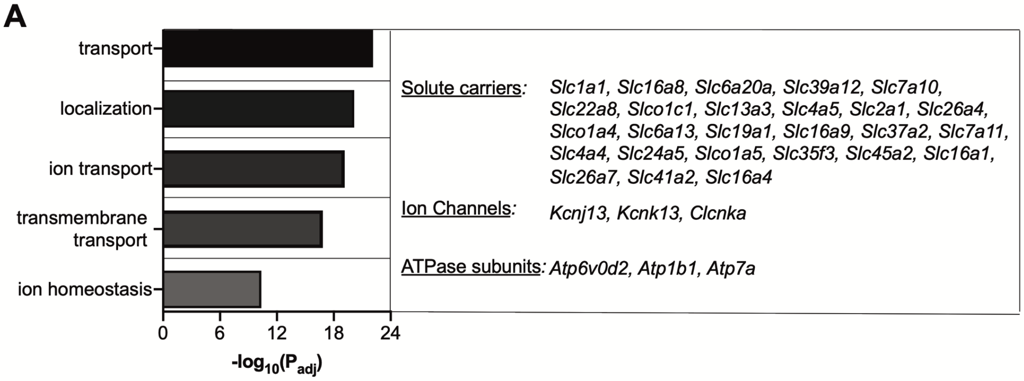

Figure S6. GO terms between VAD *Stra6*$^{-/-}$ and WT samples. The GO biological processes ontology terms in upregulated gene set (*A*) gene comparing VAD *Stra6*$^{-/-}$ relative to VAD WT.
